# Supplementary material for: From sequence to enzyme mechanism using multi-label machine learning
Source: BMC Bioinformatics. 2014 May 19;15:150. doi: 10.1186/1471-2105-15-150 (PMC4229970; doi:10.1186/1471-2105-15-150)
Supplement: Additional file 2 — Java code of ml2db. Additional file ml2db_code.tar.gz contains the Java source code to run the multi-label machine learning experiments and save the results to database. The code’s Javadoc is included. [file 1471-2105-15-150-S2.zip › additional file 2/ml2db/ml2db/doc/main/ml2db/al/database/package-tree.html]

main.ml2db.al.database Class Hierarchy


JavaScript is disabled on your browser.


- Overview
- Package
- Class
- Use
- Tree
- Deprecated
- Index
- Help

- Prev
- Next

- Frames
- No Frames

- All Classes

# Hierarchy For Package main.ml2db.al.database

Package Hierarchies:

- All Packages

## Class Hierarchy

- java.lang.Object
  - uk.ac.standrews.utils.main.database.DbManaged
    - main.ml2db.al.database.AlDataSaver
    - uk.ac.standrews.utils.main.database.DbCreator
      - main.ml2db.al.database.AlDbCreator
  - uk.ac.standrews.utils.main.database.Table
    - main.ml2db.al.database.AlEvaluationTable
    - main.ml2db.al.database.AlExperimentsTable
    - main.ml2db.al.database.QueryTable
    - main.ml2db.al.database.UtilityTable

- Overview
- Package
- Class
- Use
- Tree
- Deprecated
- Index
- Help

- Prev
- Next

- Frames
- No Frames

- All Classes
